# The divergent effects of moderate climate warming on the gut microbiota and energetic state of cold-climate lizards from open and semi-closed microhabitats

**DOI:** 10.3389/fmicb.2022.1050750

**Published:** 2022-11-22

**Authors:** Wanli Liu, Jing Yang, Yu Meng, Danyang Wu, Luoxin Cui, Teng Li, Baojun Sun, Peng Liu

**Affiliations:** ^1^College of Life Science and Technology, Harbin Normal University, Harbin, Heilongjiang, China; ^2^Key Laboratory of Animal Ecology and Conservation Biology, Institute of Zoology, Chinese Academy of Sciences, Beijing, China; ^3^College of Resources and Environmental Sciences, Nanjing Agricultural University, Nanjing, China

**Keywords:** climate warming, physiology, gut microbiota, fitness-related traits, metabolism, sympatric lizards

## Abstract

**Introduction:**

Understanding the physiological responses to warming temperatures is critical for evaluating the vulnerabilities of animals to climate warming. The physiological responses are increasingly affected by gut microbiota. However, the interactions between physiological responses and the gut microbiota of sympatric animals from various microhabitats in the face of climate change remain largely unknown.

**Methods:**

To evaluate the effects of warming temperatures on animals from different microhabitats, we compared locomotor performance, metabolic rate, growth, survival, and gut microbiota of two sympatric ectothermic species (*Eremias argus* and *Takydromus amurensis*) from open and semi-closed microhabitats under present and moderate warming climate conditions, respectively.

**Results and discussion:**

We found that locomotor performance and growth rates of snout-vent length (SVL) were enhanced in both lizard species by warming climate. Interestingly, warming temperatures enhanced resting metabolic rates (RMR) in the open-habitat lizard, *E. argus*, but depressed them in the semi-closed habitat lizard, *T. amurensis*. Reversely, the metabolism-related gut microbiota was not affected by warming in *E. argus*, whereas it was significantly enhanced by warming in *T. amurensis*, indicating a plausible compensatory effect of the gut microbiota on the metabolic regulation of *T. amurensis*. Furthermore, warming likely improved immunity in both lizard species by significantly reducing pathogenic bacteria while increasing probiotics. This study found that high-latitude sympatric lizards from both open and semi-closed habitats were beneficial to warming temperatures by physiological modification and regulation of the gut microbiota and highlighted the importance of integrating the physiology and gut microbiota in evaluating the vulnerability of animals to climate warming.

## Introduction

Warming temperatures caused by climate change have threatened animals universally (Foden et al., 2008; Leung et al., 2020). The manner in which animals respond to warming temperatures determines their vulnerability to climate warming (Williams et al., 2008; Huey and Tewksbury, 2009; Kearney et al., 2009). Animals can respond to increasing temperatures induced by climate warming by modifying their behavioral, physiological, and life-history metrics (Huey et al., 2012; Logan et al., 2018; Maebe et al., 2021; Dematteis et al., 2022; Gomez Ales et al., 2022; Li et al., 2022; Zhang and Wang, 2022). Among various metrics, physiological responses such as metabolic rate might be the most important since it determines energetic allocation and other biological rates, which are the foundations for other responding metrics (e.g., Brown et al., 2004; Chown and Gaston, 2008; Logan et al., 2014; Bestion and Cote, 2018; Logan and Cox, 2020). For example, *Takydromus* lizards from low latitudes exhibit limited metabolic acclimation capacities at multiple biological hierarchies and in embryonic acute heat tolerance (EAHT); thus, they are predicted to be more vulnerable to climate warming (Sun et al., 2021, 2022). Adjustment of metabolic rates can regulate energy expenditure, and thus may buffer the negative effects of warming temperatures on animals (Marshall and Mcquaid, 2011; Ma et al., 2018b), or even benefit cold-climate ectotherms (e.g., Liu et al., 2022). In addition, locomotion can determine the ability of an animal in food and resources acquiring. Therefore, ectotherms will be vulnerable to climate change if their locomotion are depressed at extremely high body temperatures as thermal performance curve predicts (Gunderson and Leal, 2016).

As a regulator in physiology, immunity, and fitness of animals (Ramakrishna, 2013; Gould et al., 2018; Zhang et al., 2018; Rastelli et al., 2019), the gut microbiota is also depressed by warming temperatures. For instance, a 2–3°C increase in ambient temperatures induced a 34% loss in diversity of gut microbiota in common lizard, *Zootoca vivipara* (Bestion et al., 2017). Similarly, a severe warming environment (6.7°C on average) decreased the diversity of gut microbiota but increased pathogenic bacteria in race-runner *Eremias argus* (Zhang et al., 2022b). In contrast, the gut microbiota has been shown to benefit the host thermoregulation, immunity, metabolism, growth, development, and even social behaviors (e.g., Macke et al., 2017; Zhang and Wang, 2022), all of which can in turn regulate responses to warming temperatures (e.g., Kearney et al., 2010; Triggs and Knell, 2012; Montoya-Ciriaco et al., 2020). For example, the gut microbiota of tadpoles that experienced 3-week high temperatures (28°C) could modify the metabolism and enhance the heat tolerance of tadpoles (Fontaine et al., 2022). Therefore, how gut microbiota affects the physiological responses of the host to climate warming is complex and still largely controversial (Bestion et al., 2017; Bestion and Cote, 2018). More research is needed to comprehensively understand the relationship between physiology and gut-microbiota responses to warming temperatures, which is critical for evaluating the vulnerabilities of animals and interpreting their mechanisms.

Recently, the beneficial effects of climate warming on high-latitude animals have been increasingly demonstrated (e.g., Cui et al., 2022; Liu et al., 2022). However, the differences in the effects of warming temperatures on the interaction between physiological metrics and gut microbiota in different microhabitats are unknown (e.g., open habitats, semi-closed habitats, and shaded habitats). Animals from different microhabitats might be differently affected by a warming climate. For example, ectotherms in open habitats have higher thermal preferences and tolerances than congeners in semi-closed habitats, and thus they are therefore predicted to be less vulnerable to climate warming (Li et al., 2017; Wang et al., 2019; Gomez Ales et al., 2022). Similarly, the abundance of anaerobic gut bacteria is significantly lower in flying insects than in siblings underground (Yun et al., 2014). Therefore, sympatric animals constitute an ideal research system for investigating the different effects of climate warming on animals from different microhabitats.

In this study, we selected two high-latitude and oviparous sympatric lizards from different microhabitats (*E. argus*: open habitat; *Takydromus amurensis*: semi-closed habitat) to address the physiological and gut-microbiota, as well as their potentially interactive responses to climate warming. We first mimicked the present climate and moderate warming conditions based on natural ambient temperatures to rear the lizards; we then tested the physiological metrics of locomotion and metabolic rates; we also determined the fitness-related metrics of growth and survival. Furthermore, we applied high-throughput sequencing on 16S rRNA gene amplicons from the feces of *E. argus* and *T. amurensis* under present and warming climates to determine the diversity, composition, and functions of the gut microbiota. By relating the physiological responses and gut microbiota, we further aimed to determine the underlying mechanisms at the microbiological level that affect physiological responses to warming temperatures of high-latitude lizards from different microhabitats.

## Materials and methods

### Study system and lizard collection

The Mongolian racerunner, *E. argus*, is a small lizard [average snout-vent length (SVL) = 56.30 mm] that originated in Central Asia. It inhabits plains, hills, and other warm areas with dry sands and open environments (Supplementary Figures 1, 3). The Heilongjiang grass lizard, *T. amurensis*, is a small lacertid (average SVL = 55.30 mm) that distributes in northeastern China, near the boundary of Russia and the Korean Peninsula. It mostly inhabits a mixture of shrubland and grasslands, which are cool and semi-closed environments (Supplementary Figures 2, 3). Both *E. argus* and *T. amurensis* are from the Lacertidae, with the primary food being the larvae and adults of insects (Zhao and Adler, 1993; Zhao et al., 1999). The thermal biology and distribution of *E. argus* and *T. amurensis* indicate that they are cold-climate species (Zhao et al., 2008; Park et al., 2014; Li et al., 2017; Hao et al., 2020).

In late May 21, 2021 adult *E. argus* individuals (10 females and 11 males) (45.8°N, 126.5°E) and 40 adult *T. amurensis* individuals (32 females and 8 males) (45.2°N, 127.9°E) were captured by hand and noose in Harbin, China. During collection, three data loggers (iButton, DS1921; MAXIM Integrated Products Ltd., San Jose, CA, USA) were set in the field to collect the hourly ambient temperatures for each sample site. The collected lizards were then transferred to the laboratory and reared in semi-natural enclosures.

### Experimental design and lizard husbandry

After being transferred to the laboratory, the lizards were individually marked, measured (SVL ± 1 mm), and weighed (±0.001 g) after 1 day recovery. Then, all lizards were released into semi-natural enclosures for husbandry under different thermal treatment. Semi-natural enclosures were built at Harbin Normal University within the natural distribution range of *E. argus* and *T. amurensis*.

The enclosures for *E. argus* were using their natural substrate of sand, while the enclosures for *T. amurensis* were using the soil substrate. The vegetation in the enclosures were transferred from the site they were collected. Following established method (Liu et al., 2022), we mimicked the thermal environments under the present and warming conditions for each species. We set the temperatures in the present climate similar to the field temperatures (Supplementary Figure 3). As the moderate warming scenario are universally accepted in experimental design of global change simulation (e.g., Bestion et al., 2015, 2017; Sun et al., 2018b,^2021^; Liu et al., 2022), we set the temperatures in the warming climate mimicked a moderate climate warming scenario (Shared Socioeconomic Paths, SSP1-2.6, 1.3–2.4°C) (IPCC, 2021). We manipulated the thermal environments in the enclosures using a shaded net to simulate the present climate and plastic cover to simulate a warming climate, according to published methods (Sun et al., 2018b; Liu et al., 2022). We used four enclosures for each treatment of each species. The lizards from each species were released evenly and randomly into both present and warming climate conditions (*E. argus*: present climate vs. warming climate = 5♀ 5♂ vs. 5♀ 6♂; *T. amurensis*: present climate vs. warming climate = 16♀ 4♂ vs. 16♀ 4♂). Husbandry was conducted during the active season (i.e., summer) and lasted from June to August 2021. During husbandry, we monitored the operative temperatures (T_*e*_) hourly in the enclosures using iButtons (DS1921; MAXIM Integrated Products Ltd., San Jose, CA, USA) sealed in copper tube models. The models were evenly and randomly placed in enclosures (Liu et al., 2022). Supplementary food (larval *Tenebrio molitor* and crickets dusted with vitamins) was provided twice a week.

### Locomotor performance

**FIGURE 1:**
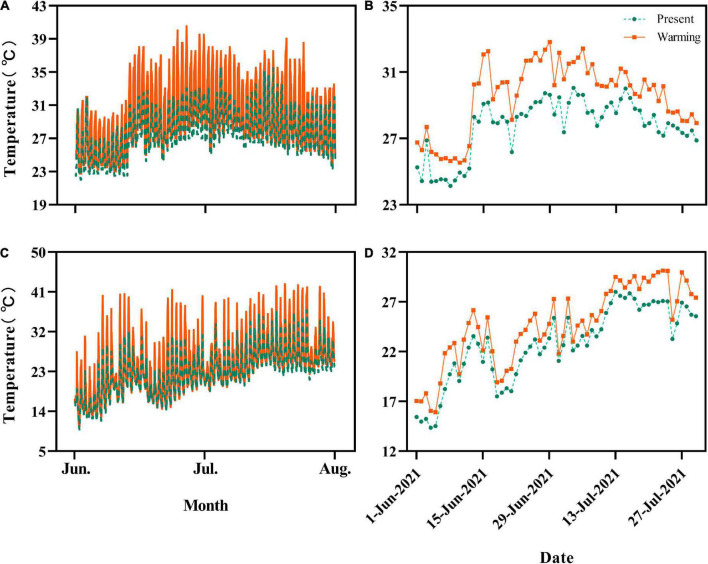
Hourly and daily average temperatures experienced by *Eremias argus* **(A,B)** and *Takydromus amurensis* **(C,D)** in the semi-natural enclosures, respectively. Green and orange lines indicate the temperatures of the present climate and warming climate conditions, respectively.

After 2-months of husbandry, sprint speed was determined as locomotor performance. Based on ambient temperatures under the present and warming climate conditions in the semi-natural enclosures, locomotor performance was measured at two test temperatures for each species in a randomized sequence (i.e., *E. argus*: 24, 32°C; *T. amurensis*: 22, 30°C; see details in Figure 1 and Supplementary Figure 3). The lizards were acclimated in an incubator at the test temperature for approximately 2 h before the test. To check whether the body temperatures of lizards matched the test temperatures, we measured the body temperatures of a subset of lizards before the test (Sun et al., 2014). Locomotor performance was tested by stimulating the lizard to run through a racetrack; that was recorded using an HD video camera (Sony, DCRSR220E, Japan). The racetrack was 1,500 × 100 × 150 mm, with intervals marked every 200 mm. Each lizard was stimulated using a paintbrush to run twice at each temperature, with an interval of 1 h for rest. The videos were analyzed using AVS Video Editor. For each lizard, the fastest speed through 200 mm was recorded for each running, and the fastest record in two running was used as the sprint speed for each lizard (Taylor et al., 2020). After sprint speed determining, the lizards were released back into the enclosures for 3 days, and their resting metabolic rates (RMR) were tested.

### The resting metabolic rates

The resting metabolic rates of the lizards were determined using a “FOXBOX” Respirometry System (Sable Systems International, Henderson, GA, USA) at two test temperatures for each species in a randomized sequence (*E. argus*: 24 and 32°C; *T. amurensis*: 22 and 30°C), based on ambient temperatures under the present and warming climate conditions in the semi-natural enclosures (see details in Figure 1 and Supplementary Figure 3).

We used a respiratory gas exchange method to evaluate the CO_2_ production rate (*V*CO_2_) as an index for the RMR following established methods (Ma et al., 2018a; Sun et al., 2020). In summary, individuals were fasted for 24 h in the laboratory before testing. At the beginning of the test, the lizards were acclimated to the test temperatures for 2 h in a temperature-controlled incubator (Blue Pard MGC-100, China). Subsequently, the test was conducted in a closed-circuit system (∼300 mL volume), with the chamber housed in an incubator. The circuit system was first opened to air and the air was scrubbed with water and CO_2_ using a tube with a flow rate of 240 mL/min to stabilize the baseline. After 5 min, the system was transferred to a closed cycle by connecting the output and the input to the tube. The carbon dioxide production rate (slope of CO_2_ volume increase) in the closed-circuit system was continuously recorded for at least 15 min, and the slope was used to calculate the RMR of the individuals. After metabolic rates determination, we measured the body mass of the lizards. Then mass-specific respiratory gas exchange (*V*CO_2_) was used to indicate metabolic rates, expressed as mL/g/h (Ma et al., 2018a; Sun et al., 2020). During the test, the lizards were kept in a chamber with dark surroundings. To minimize the effect of circadian rhythms on RMRs, tests were performed from 8 a.m. to 16 p.m.

### Growth rates

After the RMR test, the lizards were re-captured, re-measured (SVL ± 0.01 mm), and re-weighed [body mass (BM) ± 0.001 g]. No individuals died during the experiment. Therefore, the survival rate for the 2-month husbandry period was 100% for each species under both the present and warming climate conditions. Using established methods (e.g., Sun et al., 2011; Hao et al., 2021), the growth rates of SVL and BM were calculated as the daily change in SVL (mm/day) and BM (g/day).

### Feces collection and gut microbiota analysis

After the physiological metrics were tested, the feces of the lizards were started to be collected for gut microbiota analysis. To avoid inter-experiment effects, the analysis was started after putting the lizards back into the enclosure for a few days. In brief, we collected the lizards on sunny days, and transferred the lizards to the laboratory. To avoid contamination, the entire process was performed on a clean bench on the day when the lizards were collected. Because the feces of individuals were limited in volume, the feces from 3 to 6 adult individuals from the same treatment were pooled to compose one biological sample. Therefore, the sample size for each group (i.e., each species under each treatment) was evenly three. We totally pooled the feces into three biological samples for each species under each treatment. Each biological sample was placed in a sterile tube and stored at −80°C until DNA extraction.

Deoxyribonucleic acid extraction, amplification, and sequencing were conducted by PersonalBio Biotechnology Co., Ltd. (Shanghai, China). In brief, complete DNA samples were extracted using the E.Z.N.A™ Mag-Bind Soil DNA Kit (M5635, OMEGA, USA), following the manufacturer’s protocol. The quantity and quality of the extracted DNA were assessed using a fluorescence spectrophotometer (Quantifluor-ST fluorometer, Promega, E6090; Quant-iT PicoGreen dsDNA Assay Kit, Invitrogen, P7589) at 260 and 280 nm, respectively, and was also detected *via* 1.2% agarose gel electrophoresis. PCR amplification of the bacterial 16S rRNA gene V3-V4 hypervariable region was performed using a forward primer (338F:5′-ACTCCTACGGGAGGCAGCA-3′) and reverse primer (806R:5′-GGACTACHVGGGTWTCTAAT-3′). The PCR reaction (25 μL) was prepared as follows: template DNA 1 μL, amplicon PCR forward primer (10 μM) 1 μL, amplicon PCR reverse primer (10 μM) 1 μL, dNTP (2.5 mM) 2 μL, Fast Pfu DNA Polymerase (0.25 μL), 2 × buffer (5 μL), and ddH_2_O (14.75 μL). PCR was performed using the following program: denaturation at 98°C for 5 min followed by 25 cycles consisting of denaturation at 98°C for 30 s, annealing at 52°C for 30 s, and extension at 72°C for 45 s, with a final extension of 5 min at 72°C. PE250 paired-end sequencing was performed according to the Illumina MiSeq (Illumina San Diego, CA, USA) instrument manual after the DNA libraries were mixed. Raw Illumina amplicon reads were processed using the QIIME2 Core 2019.7 distribution (Bolyen et al., 2019). The Divisive Amplicon Denoising Algorithm (DADA2) pipeline that is implemented in QIIME 2 platform was used to conduct the sequence quality control, including filtering reads for quality, denoising reads, merging forward and reverse reads, removing chimeric reads, and assigning reads to amplicon sequence variants (ASVs) (Callahan et al., 2016). The ASVs were taxonomically classified using the GreenGenes database classifier v13.8, as it is universally employed and its comprehensive range of microbiota (Desantis et al., 2006). The classifier was trained to differentiate taxa present in the 99% Greengenes (v13.8) full-length reference set. Finally, a total of 388,885 and 393,639 valid sequences of the hypervariable V3-V4 region of the 16S rRNA gene were obtained from fecal samples for *E. argus* and *T. amurensis*, respectively.

### Statistical analysis

Statistical analyses were conducted using the SPSS software (version 21.0; SPSS, Inc., Chicago, IL, USA). Normality and homogeneity were evaluated using the Kolmogorov–Smirnov and *Levene’s* tests, respectively. The differences in the daily average temperatures between the present and warming climate conditions during husbandry were analyzed using a dependent *t*-test. Sprint speed and RMRs were analyzed using repeated measures ANOVAs, with thermal treatments as the main factors and test temperatures as repeated-measure factors. The growth rates in SVL and BM were analyzed using general linear models with thermal treatments as factors.

Alpha diversity indices (ACE, Shannon index) and beta diversity metrics (Bray-Curtis dissimilarity and weighted UniFrac distance) were calculated using QIIME2, with the ASVs table rarefied to 32,335 reads per sample. Principal coordinate analysis (PCoA) and non-metric multidimensional scaling (NMDS) based on the Bray-Curtis distance were used to determine the variations in community diversity among different samples (Ramette, 2007). Using the Mann–Whitney U test, we compared the changes in the relative abundance of the gut microbiota composition between the present and warming climate conditions in *E. argus* and *T. amurensis*.

The unique and shared ASVs between the groups were plotted using a Venn diagram. The linear discriminant analysis (LDA) effect size (LEfSe) method was employed to identify variations in microbial communities based on LDA sources (Segata et al., 2011). The statistical significance level was set at α = 0.05. The function of gut microbiota was predicted using PICRUSt2 (Phylogenetic Investigation of Communities by Reconstruction of Unobserved States) based on the KEGG (Kyoto Encyclopedia of Genes and Genomes) database according to 16S rRNA sequencing data, which was based on the ASV tree from the Greengene database (Langille et al., 2013). Welch’s *t*-test and false discovery rate (FDR) adjusted *p*-values were used to test the differences in genes, and KEGG pathways^1^ were compared between groups to predict their function. All data were analyzed using the Personalbio Gene Cloud.^2^

## Results

### Thermal environments experienced by lizards

The daily average temperature for *E. argus* in the warming climate conditions (29.56 ± 0.27°C, 25.53–32.81°C) was 1.84°C higher than those of the present climate conditions (27.72 ± 0.22°C, 24.14–30.04°C) (*t* = 18.436, *df* = 59, *P* < 0.0001; Figures 1A,B). Similarly, the daily average temperature for *T. amurensis* in the warming climate conditions (24.63 ± 0.51°C, 15.92–30.15°C) was 1.93°C higher than those of the present climate conditions (22.67 ± 0.49°C, 14.36–28.02°C) (*t* = 22.687, *df* = 59, *P* < 0.0001; Figures 1C,D).

### Locomotor performance

**FIGURE 2:**
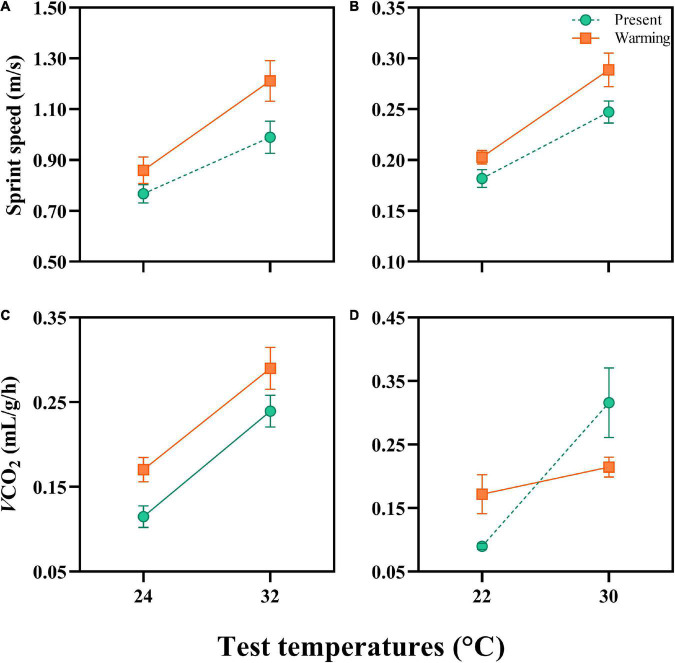
Sprint speed and resting metabolic rates (RMR) of *Eremias argus* **(A,C)** and *Takydromus amurensis* **(B,D)** from the present and warming climate conditions. The test temperatures for *E. argus* were 24 and 32°C, and for *T. amurensis* were 22 and 30°C, respectively. Green and Orange dots and lines indicate the present and warming climate conditions, respectively. Data are shown as mean ± standard error (SE).

The sprint speed of *E. argus* was significantly enhanced by test temperatures from 24 to 32°C [*F*(1,38) = 32.686, *P* < 0.0001]. Lizards in the warming climate had higher sprint speeds than those in the present climate [*F*(1,38) = 5.167, *P* = 0.029; Figure 2A]. Similarly, the sprint speed of *T. amurensis* was significantly enhanced by test temperature from 22 to 30°C [*F*(1,78) = 93.203, *P* < 0.0001]. Lizards from the warming climate had a higher sprint speed than those from the present climate [*F*(1,78) = 4.964, *P* = 0.029; Figure 2B].

### Resting metabolic rates

Resting metabolic rates was significantly enhanced by the increase in test temperatures in both species [*E. argus*: *F*(1,14) = 77.721, *P* < 0.001; *T. amurensis*: *F*(1,9) = 17.449, *P* = 0.002]. The RMR of *E. argus* was significantly enhanced in the lizards from the warming climate [*F*(1,14) = 5.171, *P* = 0.039, Figure 2C], whereas the RMR of *T. amurensis* was not affected by climate treatment [*F*(1,9) = 0.109, *P* = 0.749]. Notably, the interaction between test temperature and thermal treatment significantly influenced the RMR of *T. amurensis* [*F*(1,9) = 8.163, *P* = 0.019]. For *T. amurensis*, at a low-test temperature (22°C), RMR was higher in the warming treatment, whereas at a high-test temperature (30°C), the RMR in the warming treatment was lower than those in the present treatment (Figure 2D).

### Growth rates

**FIGURE 3:**
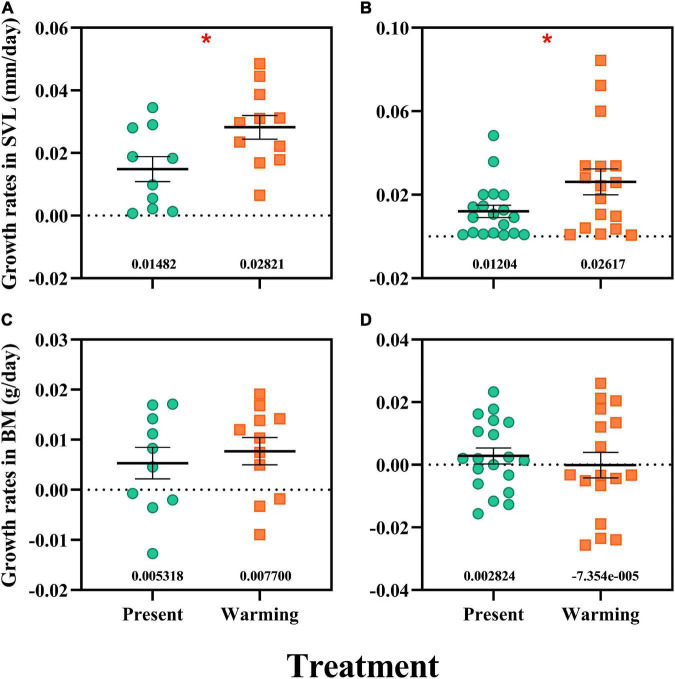
Growth rates in snout-vent length (SVL) and body mass (BM) of *Eremias argus* **(A,C)** and *Takydromus amurensis* **(B,D)**. Each dot indicates an individual; the green and orange dots indicate the present and warming climate conditions, respectively. Data are shown as mean ± standard error (SE). *Asterisk indicates significant differences between present and warming climate conditions.

Warming climate conditions significantly enhanced the growth rate (GR) in SVL for both lizard species [*E. argus*: *F*(1,19) = 5.920, *P* = 0.025, Figure 3A; *T. amurensis*: *F*(1,34) = 4.571, *P* = 0.040, Figure 3B], whereas GR in BM was not affected in either species [*E. argus*: *F*(1,19) = 0.331, *P* = 0.572, Figure 3C; *T. amurensis*: *F*(1,34) = 0.380, *P* = 0.542, Figure 3D].

### Bacterial diversity and community structure of gut microbiota

Rank abundance and rarefaction were constructed based on ASVs and showed that the depth of the sequencing results was sufficient (Supplementary Figures 4, 5). In total, we obtained 5,361 and 5,466 ASVs from the present and warming climate conditions of *E. argus* and 1,090 and 2,047 ASVs from the present and warming climate conditions of *T. amurensis*, respectively. The lizards under present and warming climate conditions shared 1,729 ASVs (Supplementary Figure 6A); however, 3,632 and 3,737 ASVs were unique to the present and warming climate conditions of *E. argus*, respectively. In addition, present and warming climate conditions shared 382 ASVs (Supplementary Figure 6B), but 708 and 1,665 ASVs were unique to the present and warming climate conditions of *T. amurensis*, respectively. Our results showed that warming did not change the bacterial community of either species (Supplementary Figure 7 and Supplementary Tables 1, 2).

### Taxonomic composition of gut microbiota

**FIGURE 4:**
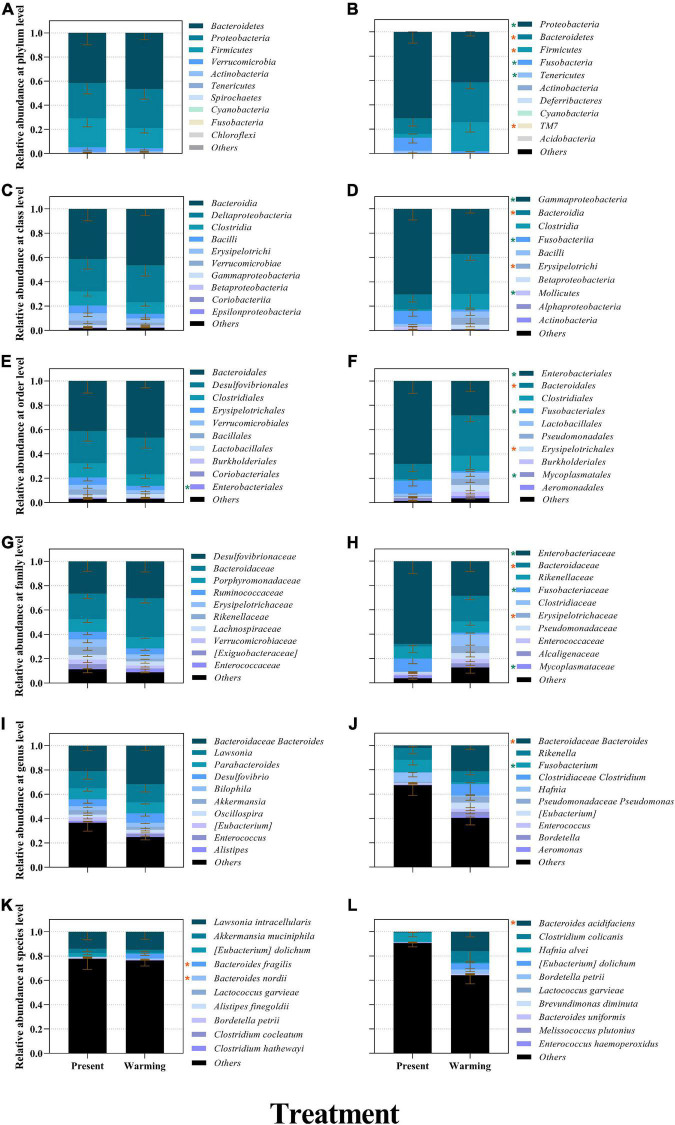
The relative abundance of bacterial compositions of *Eremias argus* **(A,C,E,G,I,K)** and *Takydromus amurensis* **(B,D,F,H,J,L)** at different levels (**A,B**: phylum; **C,D**: class; **E,F**: order; **G,H**: family; **I,J**: genus; **K,L**: species). Different colors in the figures indicate the different groups, and details are shown on the right sides of each figure, respectively. Present and warming indicate present and warming climate conditions, respectively. Data are shown as mean ± standard error (SE). Orange and green asterisks indicate significant upregulation and downregulation of warming climate conditions against the present climate conditions, respectively (*P* < 0.05).

The ASVs of *E. argus* obtained from the samples consisted of 10 phyla, 20 classes, 28 orders, 42 families, and 59 genera (Figure 4). The most dominant phyla of *E. argus* were *Bacteroidetes* (present vs. warming = 41.47% vs. 46.51%), *Proteobacteria* (present vs. warming = 29.26% vs. 32.38%), *Firmicutes* (present vs. warming = 24.07% vs. 16.87%), and *Verrucomicrobia* (present vs. warming = 3.77% vs. 2.34%) (Figure 4A). The warming climate condition significantly increased the relative abundance of *Bacteroides fragilis* and *Bacteroidales nordii* but depressed the relative abundance of the *Enterobacteriales* order and *Enterobacteriaceae* family significantly in *E. argus* (all *P* < 0.05; Figures 4E,G,K).

The ASVs of *T. amurensis* obtained from the samples consisted of 8 phyla, 16 classes, 25 orders, 42 families, and 51 genera (Figure 4). The most dominant phyla of *T. amurensis* were *Proteobacteria* (present vs. warming = 70.88% vs. 41.30%), *Bacteroidetes* (present vs. warming = 12.69% vs. 33.02%), *Firmicutes* (present vs. warming = 3.29% vs. 23.73%), and *Fusobacteria* (present vs. warming = 10.68% vs. 1.48%) (Figure 4B). The warming climate condition significantly increased the relative abundance of bacteria belonging to the phyla *Bacteroidetes*, *Firmicutes*, and *TM 7*, but significantly depressed the relative abundance of the bacteria belonging to the phyla *Fusobacteria*, *Proteobacteria*, and *Tenericutes* in *T. amurensis* (all *P* < 0.05; Figure 4B).

### Microbial community similarities and differences

**FIGURE 5:**
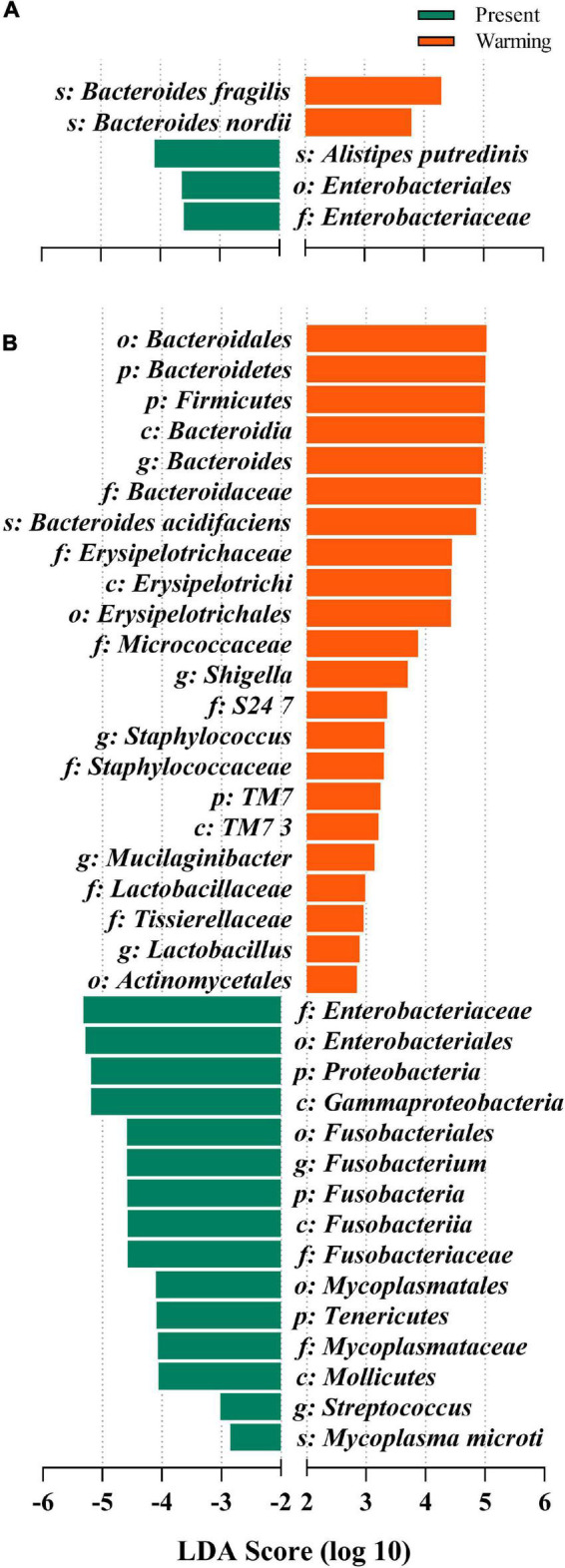
Differences in bacterial taxa determined by linear discriminative analysis of effect size (LEfSe) of **(A)** *Eremias argus* and **(B)** *Takydromus amurensis*. The highlighted taxa were significantly enriched in the group that corresponds to each color (*P* < 0.05). Linear discriminatory analysis **(**LDA**)** scores can be interpreted as the degree of difference in relative abundance. Green- and orange-colored bars indicate the present and warming groups.

Linear discriminative analysis of effect size analysis was used to detect variations in the relative abundance of microbiota at different hierarchies to further identify shifts in composition of gut microbes in different lizards. The results showed that five gut microbiota taxa differed in abundance in *E. argus*, with three taxa (1 order, 1 family, and 1 species) being more abundant in the present climate condition and two species being more abundant in the warming climate condition. The *Bacteroides* genus was the major taxon contributing to these differences (all LDA scores > 2, *P* < 0.05) (Figure 5A). In *T. amurensis*, 37 gut microbiota taxa differed in abundance, among which 15 taxa (3 phyla, 3 classes, 3 orders, 3 families, 2 genera, and 1 species) were more abundant in the present climate condition and 22 (3 phyla, 3 classes, 3 orders, 7 families, 5 genera, and 1 species) were more abundant in the warming climate condition. Three phyla, *Bacteroidetes*, *Firmicutes*, and *TM7*, were the major taxa contributing to these differences (all LDA scores > 2, *P* < 0.05) (Figure 5B).

### Prediction of bacterial functions

**FIGURE 6:**
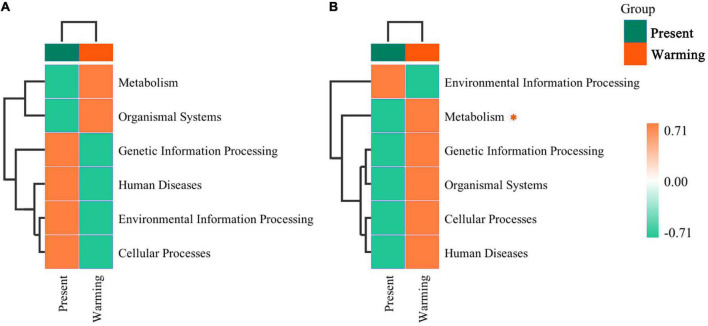
The hierarchical clustering of functional classifications in microbiota at top level between present and warming of *Eremias argus* **(A)** and *Takydromus amurensis* **(B)**. The horizontal ordinate represents the sample information and species annotation information; the cluster trees on the left and the top are species clustering and sample clustering, respectively. Gradient colors indicate the regulation of the warming climate against the present climate. *Asterisk indicates a significant difference between present and warming climate conditions (*P* < 0.05).

**FIGURE 7:**
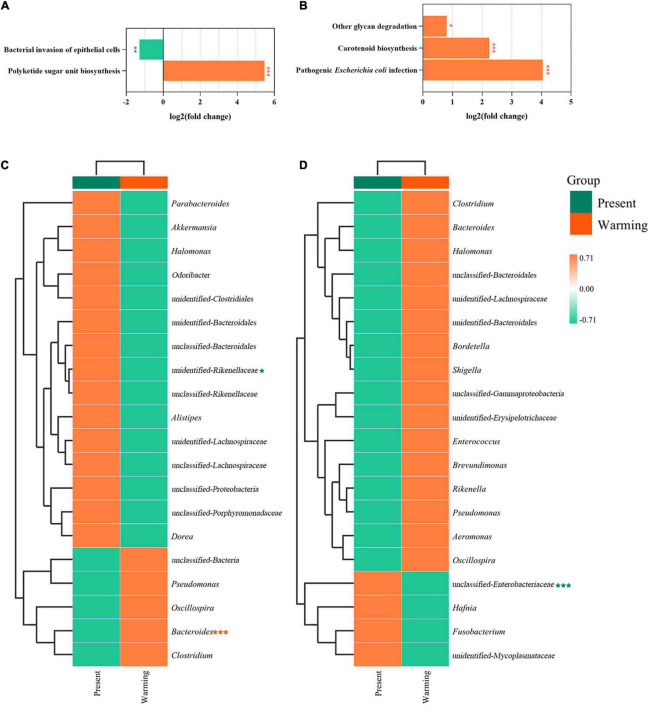
Functionally predicted KEGG pathways and the hierarchical clustering heatmap of species contribution composition of differential pathways between present and warming in *Eremias argus* **(A,C)** and *Takydromus amurensis* **(B,D)**, respectively. The horizontal ordinate represents the sample information and species annotation information; the cluster trees on the left and the top are species clustering and sample clustering, respectively. Present and warming indicate present and warming climate conditions, respectively. The highlighted pathways were significantly up-regulation (orange color) or down-regulation (green color) of warming climate against the present climate, respectively (*P < 0.05; **P < 0.01; and ***P < 0.0001).

Principal coordinate analysis analysis revealed that the function of the gut microbiota was highly aggregated in the different groups (Supplementary Figure 8A: *E. argus*; Supplementary Figure 8B: *T. amurensis*) (Supplementary Table 2). In the first-level functional classification of KEGG pathways, only the metabolic function of *T. amurensis* was significantly upregulated (*Z*-value > 0, *P* < 0.0001) (Figure 6). Upon further analysis of the differences in specific pathways, *E. argus* showed up-regulation of ko00523 (Polyketide sugar unit biosynthesis) *via Bacteroides* genus enrichment (*P* < 0.0001), and down-regulation of ko05100 (bacterial invasion of epithelial cells) through a reduction in the *unclassified-Rikenellaceae* family under warming climate conditions (*P* = 0.015) (Figures 7A,C). *T. amurensis* showed up-regulation of three pathways (ko05130-Pathogenic *Escherichia coli* infection, ko00906-Carotenoid biosynthesis, and ko00511-Other glycan degradation) caused by the reduction of the unclassified-*Enterobacteriaceae* family under warming climate conditions (*P* < 0.0001) (Figures 7B,D).

## Discussion

In this study, we mimicked thermal environments under moderate warming scenarios (SSP1-2.6,1.3–2.4°C) (IPCC, 2021) to rear cold-climate lizards (*E. argus* and *T. amurensis*) from different microhabitats and found that the effects of this moderate warming enhanced growth rate and locomotor performance but did not affect survival rates in both lizard species. These positive effects support the increasing claims that ectotherms from high latitudes would benefit from moderate climate warming (e.g., Hao et al., 2021; Cui et al., 2022; Liu et al., 2022). Interestingly, the effects of warming climate on gut microbiota differed between *E. argus* and *T. amurensis*, which are from open and semi-closed microhabitats, respectively. Although the alpha and beta diversities did not differ between the present and warming treatments in either species, the composition of gut microbiota differed between the present and warming treatments in both lizard species. According to further functional analysis, the microbiota in *T. amurensis* might be adaptive in metabolism, assisting the lizards in adapting to warming climate conditions. In addition, warming climate conditions likely improved immunity by significantly reducing pathogenic bacteria and increasing probiotics in both lizard species.

### Warming climate benefits in the physiological responses and fitness of both lizard species

Darwin fitness metrics, such as survival, growth, locomotor performance, and metabolic rates, are used universally in evaluating individual conditions and thus vulnerabilities to climate warming (e.g., Seebacher et al., 2014; Sun et al., 2014, 2018a,^2022^). For example, warming temperatures have decreased the survival rate of common lizard juveniles, thus the population was predicted to crash in the near future (Bestion et al., 2015). Similarly, spawning migration survival of Fraser River sockeye salmon (*Oncorhynchus nerka*) decreased significantly in Quesnel and Adams, British Columbia, Canada, as temperature increased, which depressed the population recruitment (Martins et al., 2011). In this study, both lizard species exhibited enhanced growth rates in the warming climate (Figure 3), but their survival rates were not affected. With the same survival, warming temperatures enhanced the growth rate, which is considered to have a beneficial effect on individuals and populations (e.g., Buckley et al., 2017; Hao et al., 2021; Liu et al., 2022). A higher growth rate allows for a larger body size before winter and enhances the survival rate over winter in ectotherms, especially for cold-climate species (e.g., Steiger, 2013; Kallis and Marschall, 2014; Lu et al., 2019). At the same time, we found enhancements in the sprint speed under a warming climate in both lizard species in this study (Figures 2A,B). Higher speeds could give lizards an advantage in activities such as predation, evading predators, and reproduction (Shu et al., 2010; Dematteis et al., 2022). In addition, metabolic rates are considered as pace regulators of life and a significant increase in the metabolic rate at high temperatures would pose a threat to tropical species (Mcnab, 2002; Dillon et al., 2010). In this study, integrating the regulation of metabolic rates in *E. argus* and *T. amurensis*, sufficient food supply, and enhancement of growth rates, we predict that the metabolic responses in both lizard species can also be beneficial. Similarly, high-latitude lizards would respond to warming temperatures by enhancing metabolic rates (i.e., *E. argus* in this study) to produce more energy with sufficient food or decreasing the metabolic rates (i.e., *T. amurensis* in this study) at high temperatures avoid excessive expenditure of energy (e.g., Sun et al., 2022). Therefore, the physiological and fitness-related responses of lizards in a warming climate revealed an advantage for lizards from both open and semi-closed microhabitats in cold climates.

### Warming climate benefit on gut microbiota in both lizard species

Alpha and beta diversities are widely used to evaluate the effects of various environmental factors on the gut microbiota of animals (e.g., Bestion et al., 2017; Macke et al., 2017; Tang et al., 2020). In this study, we found that the diversity of gut microbiota did not change significantly between the present and warming climate conditions. A similar diversity of gut microbiota across climate conditions implied that warming had a neutral effect on gut microbiota. However, the composition of the gut microbiota differed between the present and warming climate conditions for both *E. argus* and *T. amurensis*. In this study, *Bacteroidetes*, *Firmicutes*, and *Proteobacteria* were the dominant microflora in both *E. argus* and *T. amurensis* (Figures 4A,B), which was consistent with previous findings in other reptile species/populations (e.g., Ren et al., 2016; Kohl et al., 2017; Tang et al., 2020; Ibanez et al., 2021; Zhang et al., 2022b). Under warming climate conditions, the relative abundance of *Bacteroidetes* was significantly increased in *E. argus*, whereas the relative abundance of *Bacteroidetes* and *Firmicutes* was increased in *T. amurensis* (Figures 4, 5). *Bacteroidetes* and *Firmicutes* participate in the metabolism of carbohydrates, fats, and vitamins (e.g., glucose metabolism, SCFA production, and vitamin production), and play a positive role in regulating host metabolism (e.g., Colston and Jackson, 2016; Costantini et al., 2017; Lapébie et al., 2019). The regulation of the proportion of *Bacteroidetes* and *Firmicutes* may reflect the energy reserve of the body and regulate the energy balance of the body through changes in the bacterial community (Turnbaugh et al., 2006). In addition, the up-regulation of the “ko00511-Other glycan degradation” pathway in *T. amurensis* may also suggest that the warming climate has a positive effect on the metabolic function of the gut microbiota (Figures 6B, 7B,D; Martens et al., 2009; Eilam et al., 2014).

Notably, the gut microbiota of lizards in warming climate conditions also showed changes related to immunity, similar to other lizard species (e.g., Zhang et al., 2022a). In warming climate conditions, both *E. argus* and *T. amurensis* could probably enhance immunity by increasing the number of probiotics and their mediating pathways. *Bacteroides*, the main taxa that produce SFCAs (Short-chain fatty acids), have attracted much attention in recent years. In addition, it performs some immune functions (e.g., Wexler, 2007; Mazmanian et al., 2008). These functions may be related to the synthesis of antibiotics, which were related to the up-regulation of “ko00523-Polyketide sugar unit biosynthesis” of *E. argus* in the warming climate conditions (Figures 7A,C). In terms of the composition of the gut microbiota of *T. amurensis*, the warming climate conditions enhanced the relative abundance of *TM7* significantly (Figures 4B, 5B), which usually plays an active role in reducing the pathogenicity of other bacteria (e.g., Bor et al., 2019; Chipashvili et al., 2021). Meanwhile, upregulation of carotenoid biosynthesis (ko00906), which is involved in innate immunity, may also contribute to host longevity and health (Blount et al., 2003; Figure 7B). In addition, both *E. argus* and *T. amurensis* could enhance their immunity by reducing pathogenic bacteria and downregulating metabolic pathways associated with disease. We found that *Enterobacteriales* was significantly reduced in both lizard species under warming climate conditions (Figures 4E,F, 7D). As *Enterobacteriaceae* contains most of the pathogenic bacteria, its reduction can be beneficial to the health of the host under warming climate conditions (e.g., Schwab et al., 2014). The increase in carotenoid content in *T. amurensis* might have been caused by a decrease in *Enterobacteriaceae* (Figures 7B,D). *Rikenellaceae*, an inflammatory indicator, is significantly enriched when the body is under stress. Its reduction may contribute to the body’s health by interacting with the down-regulated “ko05100-Bacterial invasion of epithelial cells” of *E. argus* under warming climate conditions (e.g., Litvak et al., 2017; Brennan and Garrett, 2019; Wang et al., 2020; Figures 7A,C). Similarly, the relative abundance of pathogenic bacteria (e.g., *Fusobacteria*, *Proteobacteria*, and *Tenericutes*) in the gut microbiota of *T. amurensis* under warming climate conditions may also have a positive impact on the body’s immunity (Brennan and Garrett, 2019; Figures 4B,D,F,H,J,L). However, “ko05130-Pathogenic *Escherichia coli* infection” was also upregulated in *T. amurensis* under warming climate conditions (Figure 7B). The upregulation of conditioned pathogen-related pathways may complicate the immune response of *T. amurensis* to warming climate conditions. Therefore, we suggest that the gut microbiota of *E. argus* and *T. amurensis* responded to warming climate conditions due to changes in composition rather than diversity. Such changes are likely beneficial to the metabolism, individual health, and immunity of lizards.

### Interaction between physiology and gut microbiota facilitating the metabolism of *Takydromus amurensis* under warming climate

Interestingly, we found a plausible interaction between the metabolic rates of lizards and metabolism-related gut microbiota. When faced with a warming climate, the lizards *E. argus* from open microhabitats up-regulated their own RMRs (Figure 2C), without any regulation of metabolism-related gut microbiota (Figures 6A, 7A,C). In contrast, the lizard *T. amurensis* from semi-closed microhabitats did not regulate the metabolic rates significantly, but the metabolism-related gut microbiota was up-regulated. At high body temperatures, the RMR of *T. amurensis* was depressed due to the warming climate (Figure 2D), but the metabolism-related gut microbiota might compensate for the metabolic depression of the host, indicating a plausible complementary effect between the host and gut microbiota in the metabolic regulation of *T. amurensis* in warming climates (Figures 6B, 7B,D; O’connor et al., 2017; Gould et al., 2018; Rastelli et al., 2019).

The occurrence of this condition may be related to sufficient food and ambient temperature used in this study. As a cold-climate lizard from open habitats, *E. argus* prefers a hot environment, and sufficient food improves its metabolism under heating without negative effects (e.g., Wang et al., 2019; Sun et al., 2022). However, food is not always plentiful in natural habitats; therefore, warming may negatively affect the fitness of individuals and even populations while maintaining high metabolic rates (e.g., Bestion et al., 2015; Hao et al., 2021; Sun et al., 2022). In contrast, although the metabolism of *T. amurensis* in the semi-closed habitat was inhibited when the ambient temperature increased (Figure 2D), gut microbiota could assist energic regulation *via* composition change, which was probably beneficial for the proliferation of microbiota, which in turn assisted the thermal responses of the host (e.g., Seebacher et al., 2014; Fontaine et al., 2022). However, to comprehensively understand this complementary effect, further experimental manipulations are required to confirm the detailed regulation (e.g., Fontaine et al., 2022).

## Conclusion

In summary, we found that moderate warming climates were plausibly beneficial to lizards from both open and semi-closed microhabitats at high latitudes improving their growth rate, locomotion, and allowing for a high survival rate. Notably, we found a likely complementary effect between the host metabolic rates and the metabolism regulation of gut microbiota: *E. argus* from open microhabitats only responded *via* physiology, whereas *T. amurensis* from a semi-closed microhabitat showed an interactive effect of physiology and gut microbiota responses in addition to physiological response. Furthermore, the compositional changes in gut microbiota without any diversity change might enhance the immunity of lizards under warming climates. We encourage future experimental manipulations to determine the potential interactive and complementary effects of metabolism regulation by the physiological response of the host and the contribution of the gut microbiota. In addition, experimental verification of the enhancement of immunity by the gut microbiota is required. These manipulations are helpful in evaluating the vulnerabilities of animals to climate warming and reveal the underlying mechanisms at the microbiological level.

## Data availability statement

The datasets presented in this study can be found in online repositories. The names of the repository/repositories and accession number(s) can be found in the article/Supplementary material.

## Ethics statement

The animal study was reviewed and approved by the Animal Ethics Committees at the Institute of Zoology, Chinese Academy of Sciences (IOZ14001).

## Author contributions

PL, BS, WL, TL, and YM designed the study. WL, JY, YM, DW, and LC data collected and analyzed. JY, BS, TL, WL, and PL wrote the manuscript. All authors contributed critically to the drafts and revisions and gave final approval for publication.

## Footnotes

1
http://picrust.github.io/picrust/

2
https://www.genescloud.cn
