# Supplementary material for: The divergent effects of moderate climate warming on the gut microbiota and energetic state of cold-climate lizards from open and semi-closed microhabitats
Source: Front Microbiol. 2022 Nov 22;13:1050750. doi: 10.3389/fmicb.2022.1050750 (PMC9722725; doi:10.3389/fmicb.2022.1050750)
Supplement: Supplementary file 2 [file Data_Sheet_1.docx]

**Supplementary information**

**Table S1 The summary of alpha diversity indexes.** The data are shown as mean ± standard error (SE), and minimum to a maximum of the values. Present and warming indicate present and warming climate conditions respectively.

|  | ***Eremias argus*** | | | ***Takydromus amurensis*** | | |
| --- | --- | --- | --- | --- | --- | --- |
|  | **Present** | **Warming** | ***P* value** | **Present** | **Warming** | ***P* value** |
| **Chao1** | 2525.40 ± 436.22 | 2801.46 ± 120.79 | 0.51 | 533.41 ± 88.74 | 922.21 ± 186.40 | 0.13 |
|  | (2042.12-3396.08) | (2628.93-3034.18) |  | (433.68-710.41) | (550.14-1128.39) |  |
| **Observed species** | 2313.93 ± 347.64 | 2497.97 ± 128.45 | 0.51 | 489.33 ± 78.42 | 833.10 ± 173.82 | 0.13 |
|  | (1948.60-3008.90) | (2263.70-2706.40) |  | (403.00-645.90) | (486.70-1031.70) |  |
| **Pielou e** | 0.73 ± 0.03 | 0.75 ± 0.02 | 0.28 | 0.57 ± 0.04 | 0.60 ± 0.03 | 0.51 |
|  | (0.68-0.78) | (0.73-0.78) |  | (0.53-0.64) | (0.54-0.64) |  |
| **Shannon** | 8.15 ± 0.46 | 8.43 ± 0.25 | 0.51 | 5.05 ± 0.30 | 5.78 ± 0.47 | 0.28 |
|  | (7.46-9.03) | (8.13-8.93) |  | (4.58-5.61) | (4.86-6.41) |  |
| **Simpson** | 0.97 ± 0.01 | 0.98 ± 0.01 | 0.83 | 0.92 ± 0.02 | 0.93 ± 0.01 | 0.51 |
|  | (0.95-0.99) | (0.97-0.99) |  | (0.90-0.95) | (0.90-0.95) |  |

**Table S2 The statistical analysis of beta diversity.**

| **Adonis test** | ***F*** | **Adjusted *P* value** | ***R*^2^** |
| --- | --- | --- | --- |
| *Eremias argus* | 1.440 | 0.198 | 0.264 |
| *Takydromus amurensis* | 2.705 | 0.111 | 0.403 |


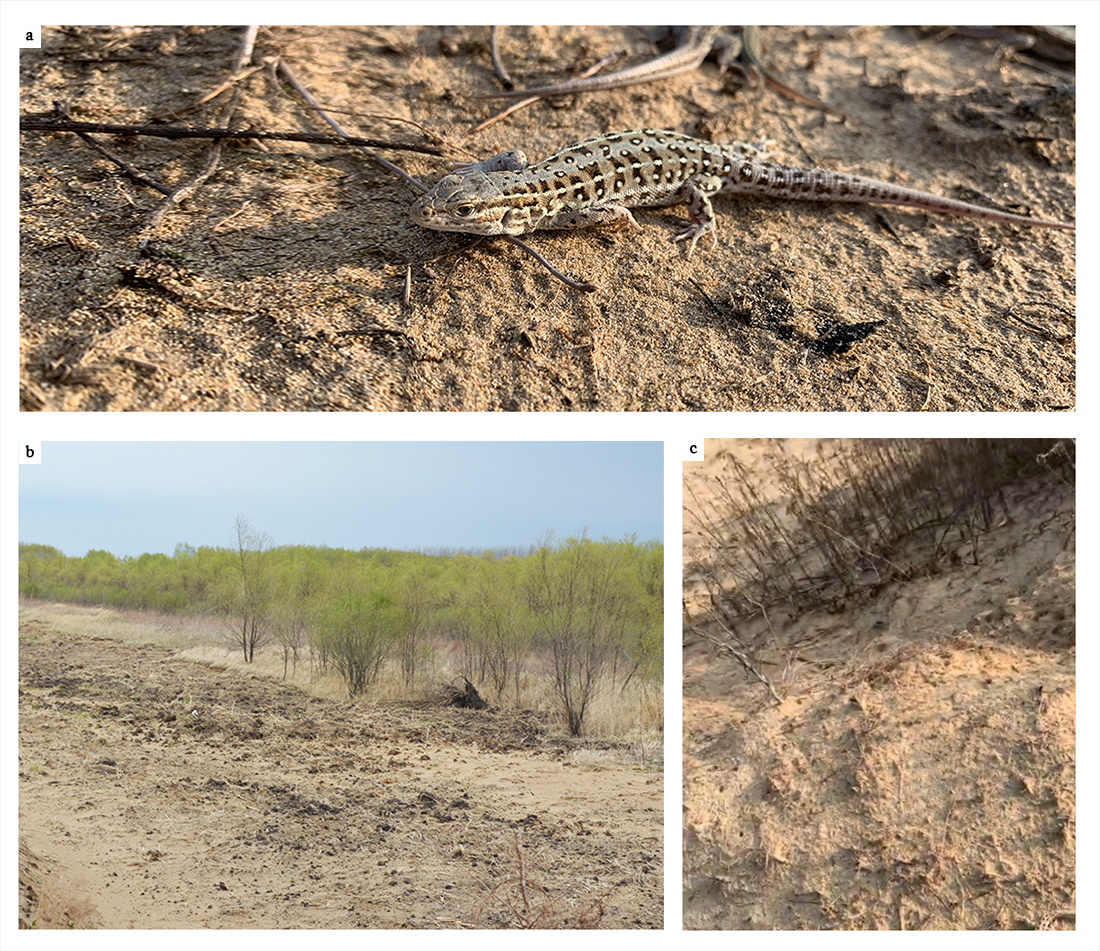


**Fig. S1 Photography of *Eremias argus* (a) and its natural habitat (b, c) at Harbin.**


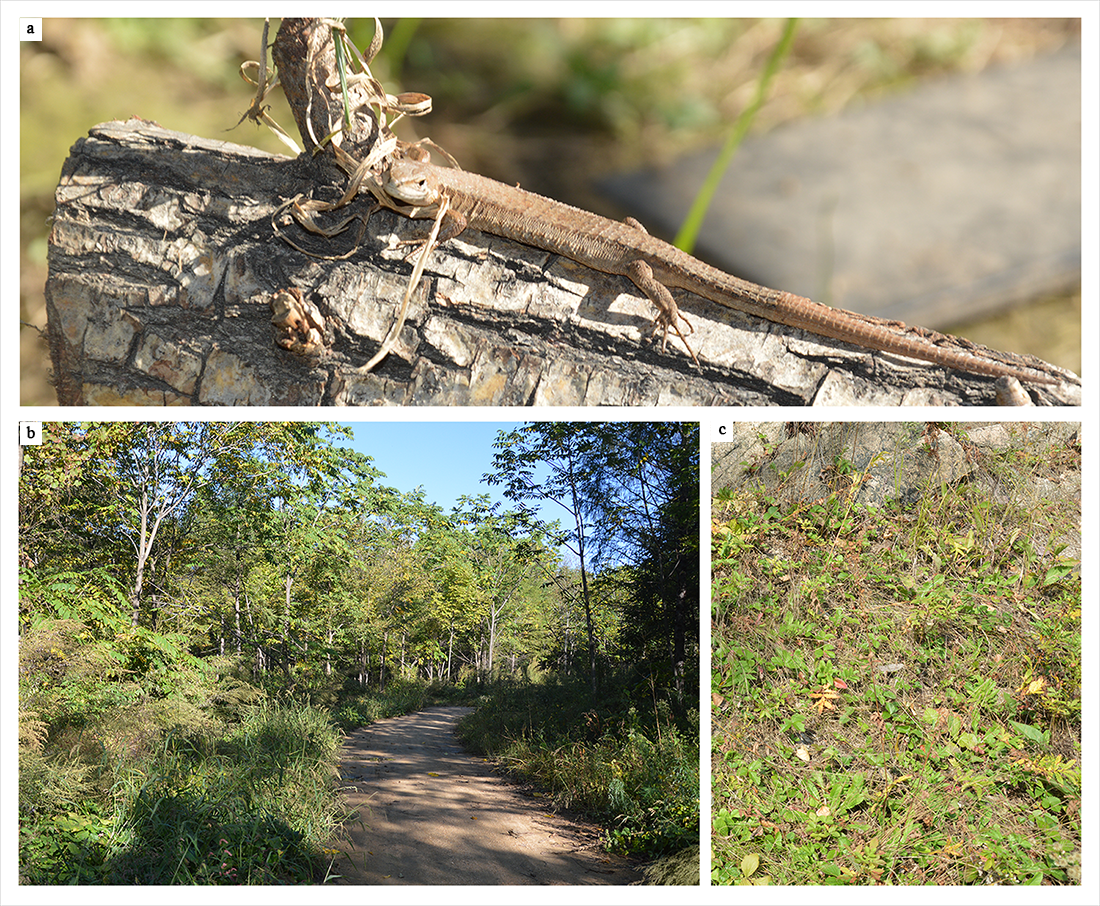


**Fig. S2 Photography of *Takydromus amurensis* (a) and its natural habitat (b, c) at Harbin.**


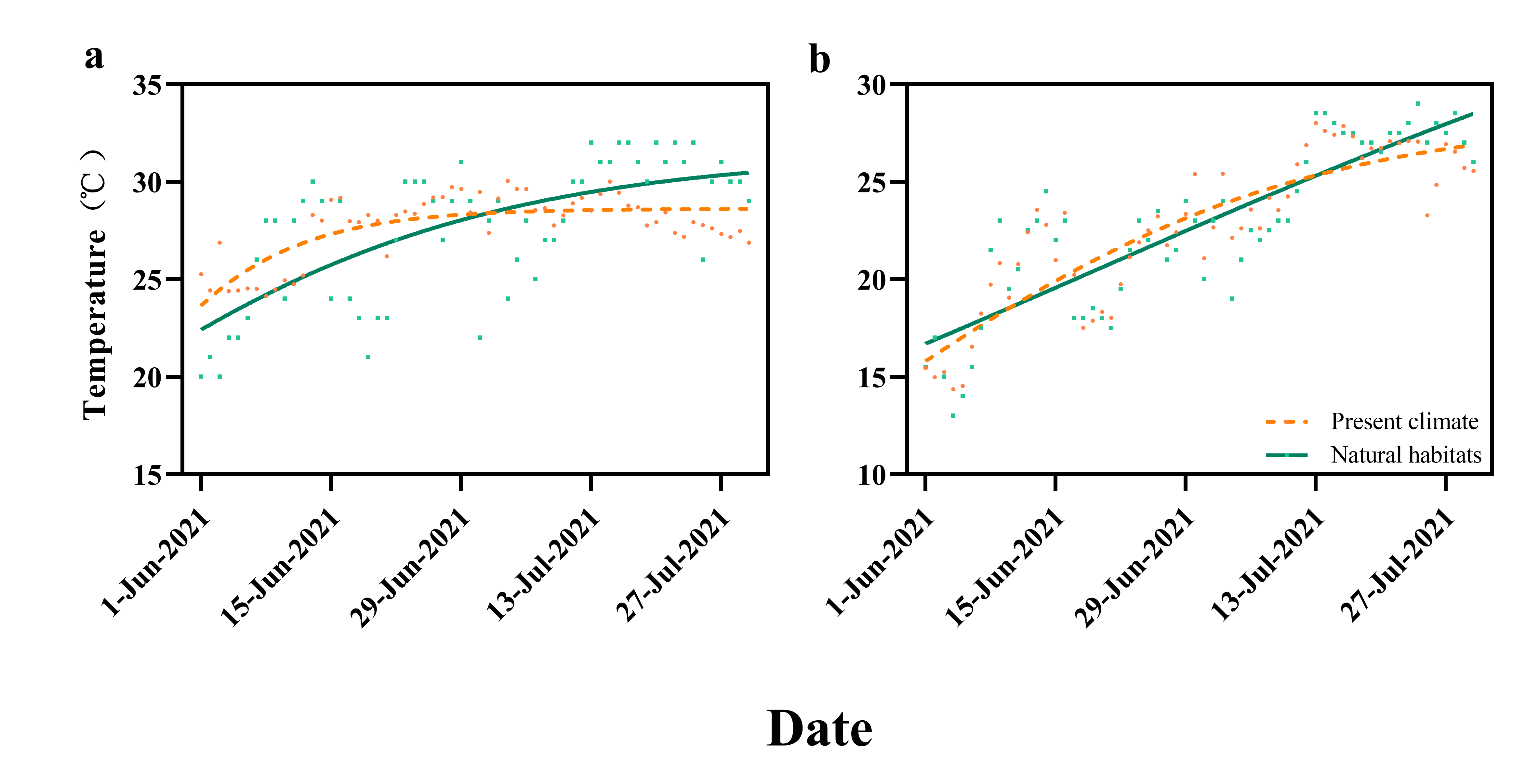


**Fig. S3 Daily average temperatures for *Eremias argus* (a) and *Takydromus amurensis* (b) in the field and the present-climate enclosures.** The orange and green lines indicate daily average temperatures for present-climate enclosures and natural habitats, respectively. The average daily temperatures from June to July are equal (natural habitats *vs.* present climate for *E. argus*: 27.60 ± 0.45 *vs.* 27.72 ± 0.22; *t* = 0.282, *df* = 59, N = 120, *P* = 0.779; for *T. amurensis*: 22.72 ± 0.54 *vs.* 22.67 ± 0.49; *t* = 0.308, *df* = 59, N = 120, *P* = 0.759). The similar daily average temperatures for the natural habitats and present-climate enclosures indicate the setup of thermal environments in our semi-natural enclosures is available to reflect the natural thermal environments.


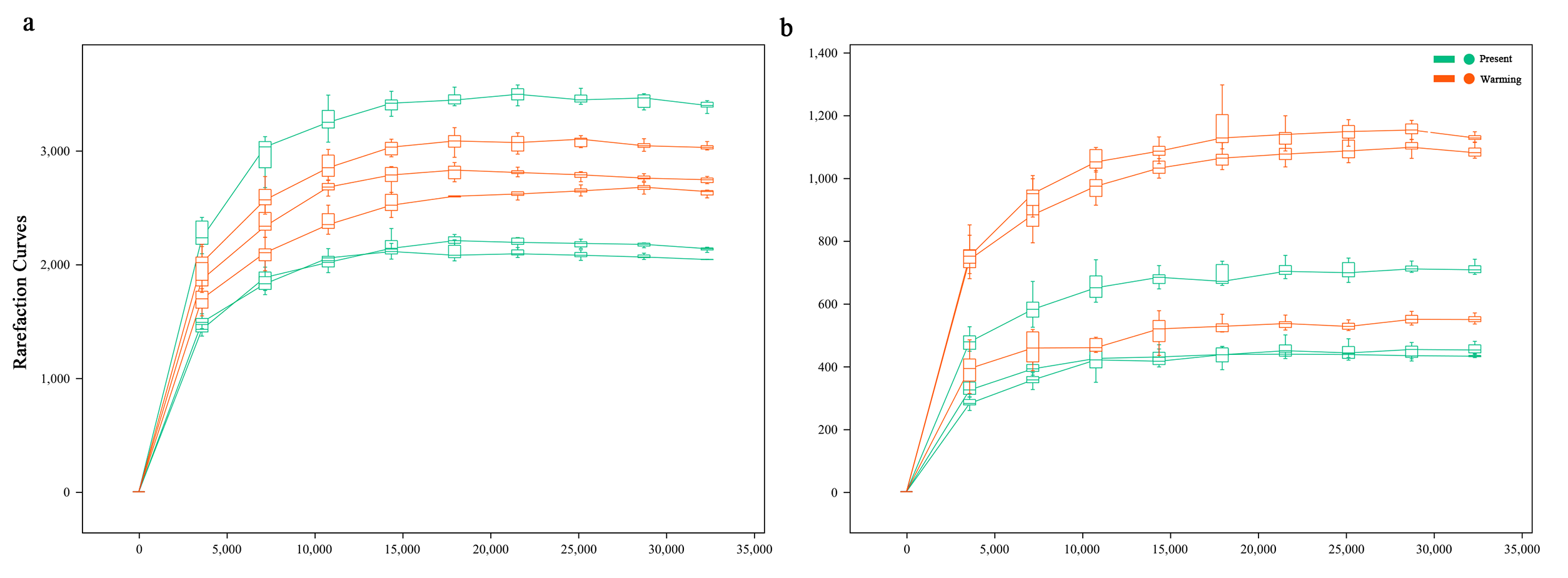


**Fig. S4 Rarefaction Curve of *Eremias argus* (a) and *Takydromus amurensis* (b) under the present and warming climate conditions.** Each green and orange line represents a mixed sample under present and warming conditions, respectively.


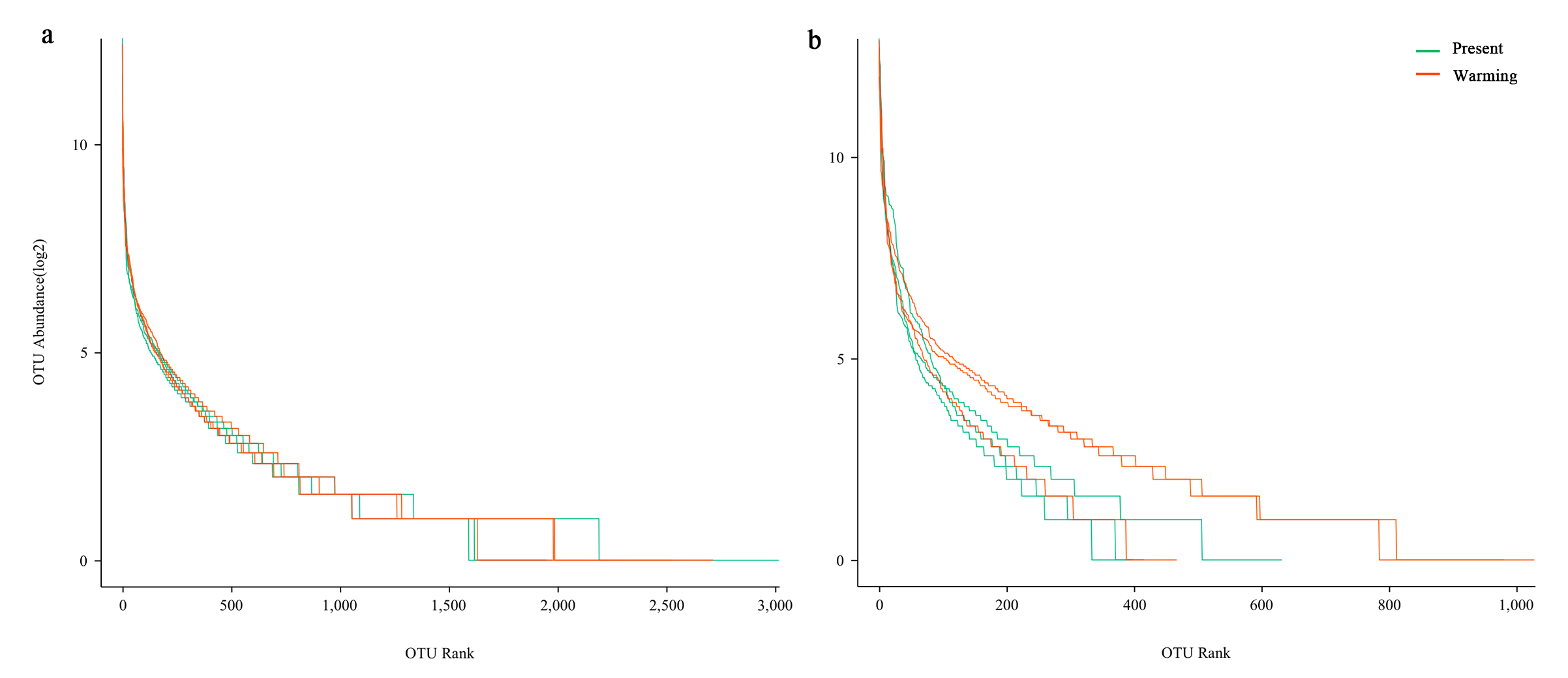


**Fig. S5 Rank abundance curve of *Eremias argus* (a) and *Takydromus amurensis* (b) under the present and warming climate conditions.** Each green and orange line represents a mixed sample under present and warming conditions, respectively.


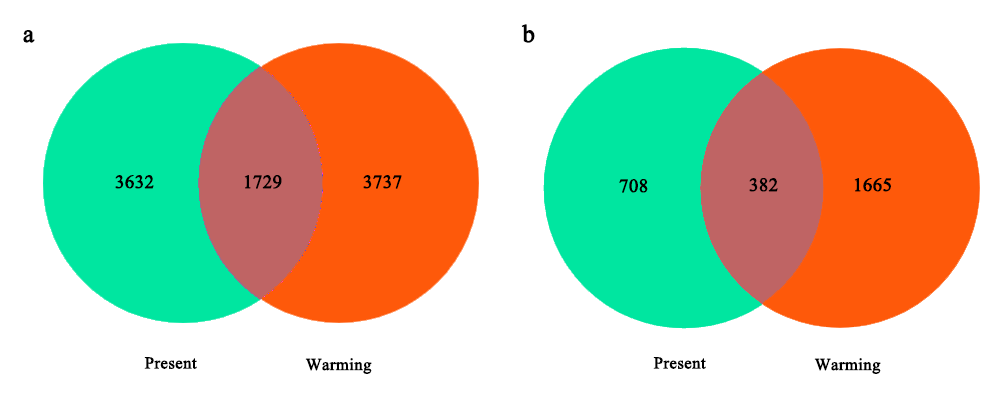


**Fig. S6 The Venn plot to show the unique and shared ASVs between present and warming climate conditions in (a) *Eremias argus* and (b) *Takydromus amurensis*.** Present and warming indicate the present and warming climate conditions respectively. The number in the areas indicate the number of ASVs.


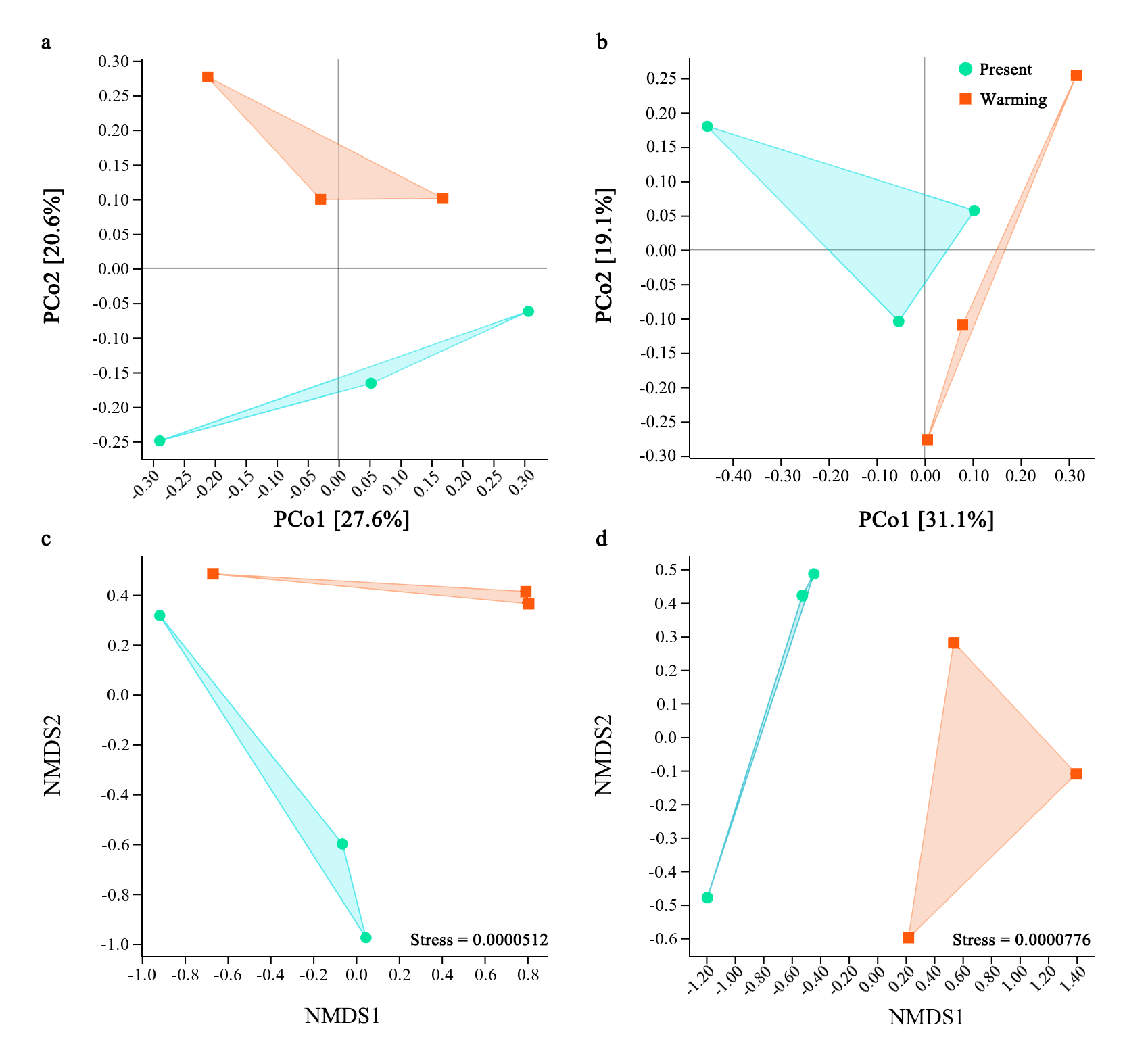


**Fig. S7 PCoA and NMDS analysis for (a, c) *Eremias argus* and (b, d) *Takydromus amurensis* based on the Bray-Curtis distance of intestinal flora.** Present and warming indicate the present and warming climate conditions respectively. Each point in the graph represents a mixed sample, the distance between points indicates the degree of difference, samples in the same group are represented in the same color. In (c, d), when the stress < 0.2, NMDS can accurately reflect the differences between groups and within groups of samples.


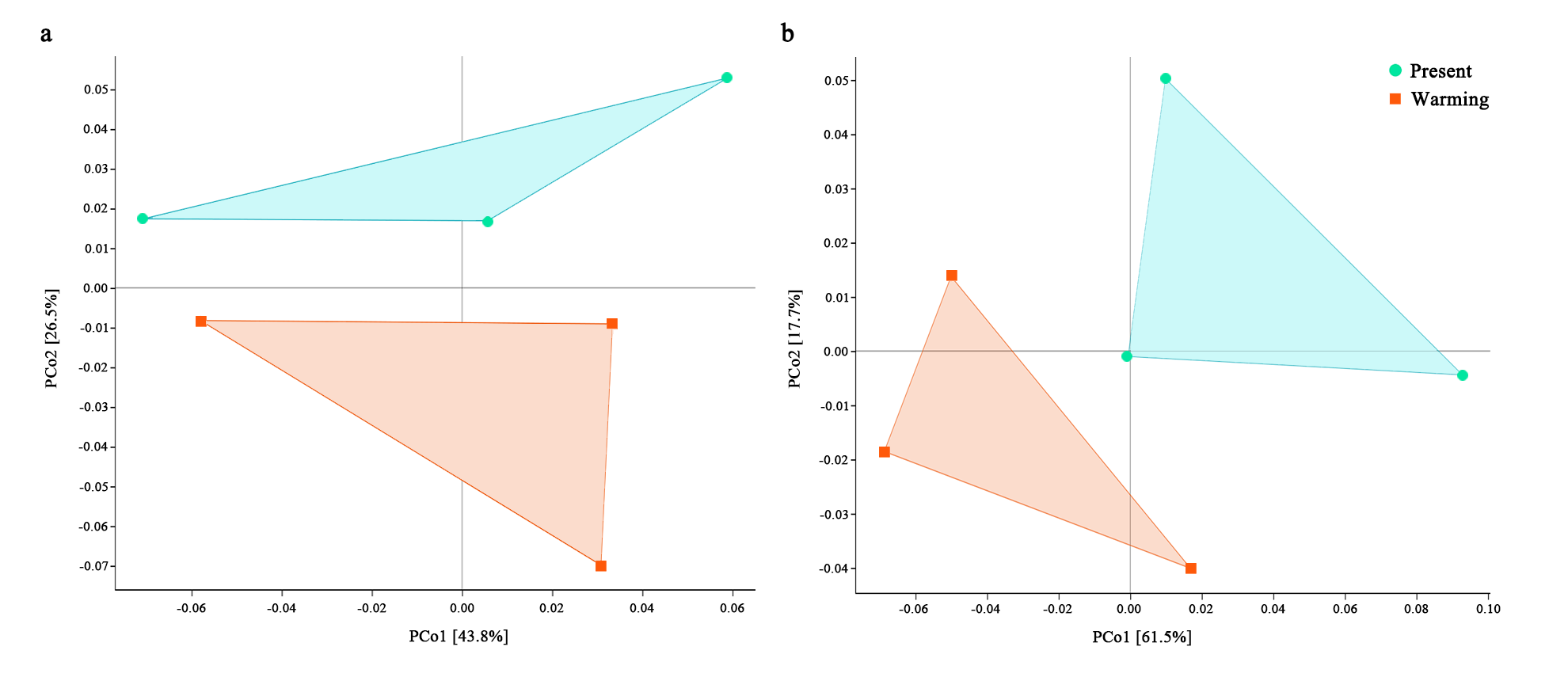


**Fig. S8 PCoA analysis of functional classification in (a) *Eremias argus* and (b) *Takydromus amurensis*.** Present and warming indicate present and warming climate conditions respectively. Each point in the graph represents a mixed sample, the distance between points indicates the degree of difference, samples in the same group are represented in the same color.
